# Post‐exercise hypotension in male spontaneously hypertensive rats: The issue of calculation method

**DOI:** 10.14814/phy2.15524

**Published:** 2023-02-17

**Authors:** Amandine Krzesiak, Julie L. Lavoie, Stéphane Sebille, Christian Cognard, Laurent Bosquet, Nathalie Delpech

**Affiliations:** ^1^ Laboratoire MObilité, Vieillissement et Exercice; EA 6314, Faculté des Sciences du Sport Poitiers France; ^2^ Laboratoire Signalisation & Transports Ioniques Membranaires ERL CNRS/Université de Poitiers no 7368 Poitiers France; ^3^ Centre de recherche du Centre Hospitalier de l'Université de Montréal (CRCHUM) Montréal Canada; ^4^ École de kinésiologie et des sciences de l'activité physique Université de Montréal Montréal Canada

**Keywords:** blood pressure variability, hypertension, physical exercise, telemetry

## Abstract

In spontaneously hypertensive rats, exercise can lead to a post‐exercise decrease in blood pressure, named post‐exercise hypotension (PEH). This can be following physical training but also after a single bout of mild to moderate exercise when measured with tail‐cuff or externalized catheter methods. Our aim was to assess the PEH obtained with different calculation methods and to compare the magnitude of this effect induced by a moderate‐intensity continuous exercise or a high‐intensity intermittent exercise. Thirteen 16‐week‐old male spontaneously hypertensive rats performed two types of aerobic exercise (continuous or intermittent) on a treadmill. Arterial pressure was recorded by telemetry for 24 h which was started 3 h before physical exercise. Based on the literature, PEH was first evaluated with two different baseline values, and then with three different approaches. We observed that the identification of PEH depended on the method used to measure the rest value, and that its amplitude was also influenced by the calculation approach and the type of exercise performed. Hence, the calculation method and the amplitude of the detected PEH can significantly influence their physiological and pathophysiological inferences.

## Graphical abstract

In spontaneously hypertensive rats, exercise can lead to a post‐exercise decrease in blood pressure recording by radio‐telemetry, nammed post‐exercise hypotension. Hence, the choice of the calculation method and the amplitude of the detected hypotension can greatly influence their physiological and pathophysiological inferences

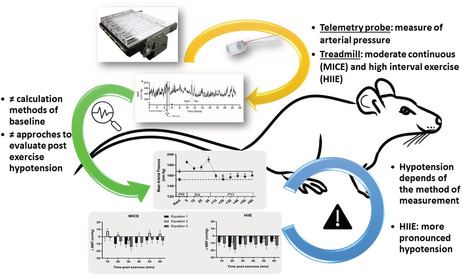

## 1 INTRODUCTION

Hypertension is a major public health problem because of its high prevalence (over 1 billion people worldwide) (Marik & Rivera., 2011) and its associated risk of cardiovascular and cerebrovascular diseases (Rapsomaniki et al., 2014), (Meissner., 2016). Considering its acute and chronic effect on the central and peripheral factors controlling blood pressure (BP), physical exercise represents a well‐recognized non‐pharmacological intervention in the prevention and treatment of hypertension and it is a cornerstone in cardiac rehabilitation (Ambrosetti et al., 2020).

More specifically, it is well established that a single bout of exercise induces a decrease in BP in hypertensive humans (Gomes Anunciacao & Doederlein, 2011) and animals (Lee et al., 2009; Lizardo, Silveira, Vassallo, & Oliveira, 2008). This so‐called post‐exercise hypotension (PEH) has been reported to start a few minutes after exercise cessation and can persist for up to 10 h (Melo, Alencar Filho, Tinucci, Mion Jr, & Forjaz, 2006). In humans, the exact magnitude and duration of this phenomenon are still open to debate, since PEH depends on the characteristics of the population [i.e., sex, age, baseline BP; (Atkinson, Cable, & George, 2005; Taylor et al., 2010)], the measurement method [i.e., oscillometric or ausculatory (Casonatto, Goessler, Cornelissen, Cardoso, & Polito, 2016)] but also the characteristics of exercise, including its intensity, duration, modality (i.e., cardiovascular or neuromuscular, intermittent or continuous, aquatic or on dryland). For example, Anunciacao and Polito (Gomes Anunciacao & Doederlein, 2011) reported a more pronounced and more prolonged PEH in hypertensive patients performing a cardiovascular exercise compared to a neuromuscular exercise. If we focus on this specific type of exercise (i.e., cardiovascular), Jones, George, Edwards, and Atkinson (2007) highlighted a dose–response relationship between exercise intensity and the magnitude and duration of PEH in hypertensive patients performing a continuous exercise. Another study (Sosner et al., 2016) reported a similar dose–response relationship in hypertensive patients performing a high‐intensity intermittent exercise versus moderate‐intensity continuous exercise. The latter finding has recently been confirmed by Pimenta et al. (2019) in the same population. Sosner et al. (2016) also reported an interaction with the environment since the magnitude and duration of PEH were increased when exercise was performed in the water compared with dry land.

Post‐exercise hypotension has also been studied in animal models. In spontaneously hypertensive rats (SHR), PEH has been reported after a neuromuscular (Faria Tde et al., 2010) and a cardiovascular exercise (Lee et al., 2009; Reger, Kolwicz, & Libonati, 2012) whether performed on a treadmill (low or medium intensity) or in a wheel (free intensity). As in humans, the PEH magnitude depends on the exercise characteristics (MacDonald, 2002), but also on the animal's characteristics, including their age and, more particularly, their sex. For example, Chandler, Rodenbaugh, and DiCarlo (1998) reported delayed onset of PEH in female vs male SHR after a moderate intensity continuous exercise performed on a treadmill. Regarding neuromuscular exercise, Dias et al. (2017) did not report PEH in female SHR, while it was observed in males (Faria Tde et al., 2010).

The influence of exercise and population characteristics on the magnitude and duration of PEH is now well recognized, both in humans and animals. If additional studies are required to better understand the exact mechanisms contributing to these differences and the way to account for them in hypertensive patients, there are also methodological considerations that contribute to this heterogeneity which are clearly underestimated. For instance, an important factor is the calculation method used to assess the PEH. Fecchio et al. underscored that the occurrence and the magnitude of PEH and its hemodynamic determinants were method‐dependent (Fecchio, Brito, Pecanha, & Forjaz, 2020).

In this context, the purpose of this study was twofold. First, we aimed to compare the results obtained with different calculation methods to identify the most reliable method using a gold‐standard BP measurement method, radiotelemetry. The second objective of this study was to use this method to compare the magnitude of PEH induced by moderate‐intensity continuous exercise (MICE) or high‐intensity intermittent exercise (HIIE) in SHR males.

## 2 MATERIALS AND METHODS

### 2.1 Animal care

Our study is a random crossover trial on 13 male spontaneously hypertensive rats (SHR) with a mean age of 16 weeks. SHR were obtained from Charles River Laboratories (weight: 300‐350 g). Animals were housed individually, with access to standard rat chow (Teklad, #2918) and water ad libitum. They were kept on a 12‐h light/dark reverse cycle (dark: 8 am to 8 pm) to perform exercise during their period of activity (awake period). Their care met the standards set forth by the Canadian Council on Animal Care for the use of experimental animals. All the procedures were approved by the university animal care committee of the Centre Hospitalier de l'Université de Montréal research center.

### 2.2 Arterial pressure measurement

#### 2.2.1 Implantation of transmitters

After 10 days of acclimatization to our animal facility, a telemetry probe (C50‐PT, Data Sciences International, St Paul, MN, USA) was implanted in each animal. Anesthesia was induced through inhalation of 4% isoflurane and then maintained with 2% isoflurane. A drop of ophthalmic lubricant was applied to each eye. The abdomen and left groin were shaved and then cleaned with 70% alcohol and chlorhexidine. An incision was made at the middle level of the abdominal wall and another along the left inner thigh to expose the femoral artery. The implant body was placed in the peritoneal cavity. The catheter tip was introduced through an incision in the femoral artery advancing 3 centimeters to obtain measurements from the abdominal aorta. The abdominal muscle wall was then sutured, and the skin was closed with absorbable sutures. Animals returned to their cage for recovery for 10 days. On the day of the surgery and the two following days, animals received carprofen (5 mg/kg), administrated by subcutaneous injection.

#### 2.2.2 Monitoring of blood pressure

During a one‐day experimental session, BP was recorded over 24 h. The frequency of acquisition varied throughout the day. BP was first measured every 20 s for 7 h to obtain a finer definition closer to the exercise period and then every minute for 17 h.

### 2.3 Exercise testing

#### 2.3.1 Habituation

Animals were placed on a treadmill for habituation 1 week before telemetry probe implantation and 1 week after to improve the reperfusion of the hind limb. Before telemetry probe implantation, the habituation period started at a speed of 15 m min^−1^ for 3 min without a slope. The slope was then increased by 5 degrees every 2 days, while the speed was increased by 2 m min^−1^ and the time by 2 min daily until the day of the surgery. Two days after surgery, we started the habituation period on a treadmill at a speed of 7 m·min^−1^ for 2 min without a slope. The slope was then augmented by 5 degrees every 2 days and the speed by 3 m·min^−1^ every day, while the duration remained the same. The duration of each habituation period was 6 days.

#### 2.3.2 Maximal continuous graded exercise test

Two days after the last habituation period (post‐telemetry probe implantation), a maximal continuous graded exercise test was performed on a treadmill. The grade was set at 10% throughout the test, with an initial velocity of 13 m min^−1^. Velocity was increased by 3.6 m min^−1^ every 2 min until the animals were unable to sustain the required intensity. To ensure that the animals ran at their maximal speed, bursts of high‐pressure air were used as an aversive stimulus. The velocity attained at the last completed stage of the test was considered the peak treadmill velocity (PTV). Two tests were carried out at a two‐day interval to account for animal variability. The highest PTV of these two tests was selected to adjust exercise intensity during the subsequent MICE and HIIE sessions.

#### 2.3.3 Exercise sessions

**FIGURE 1:**
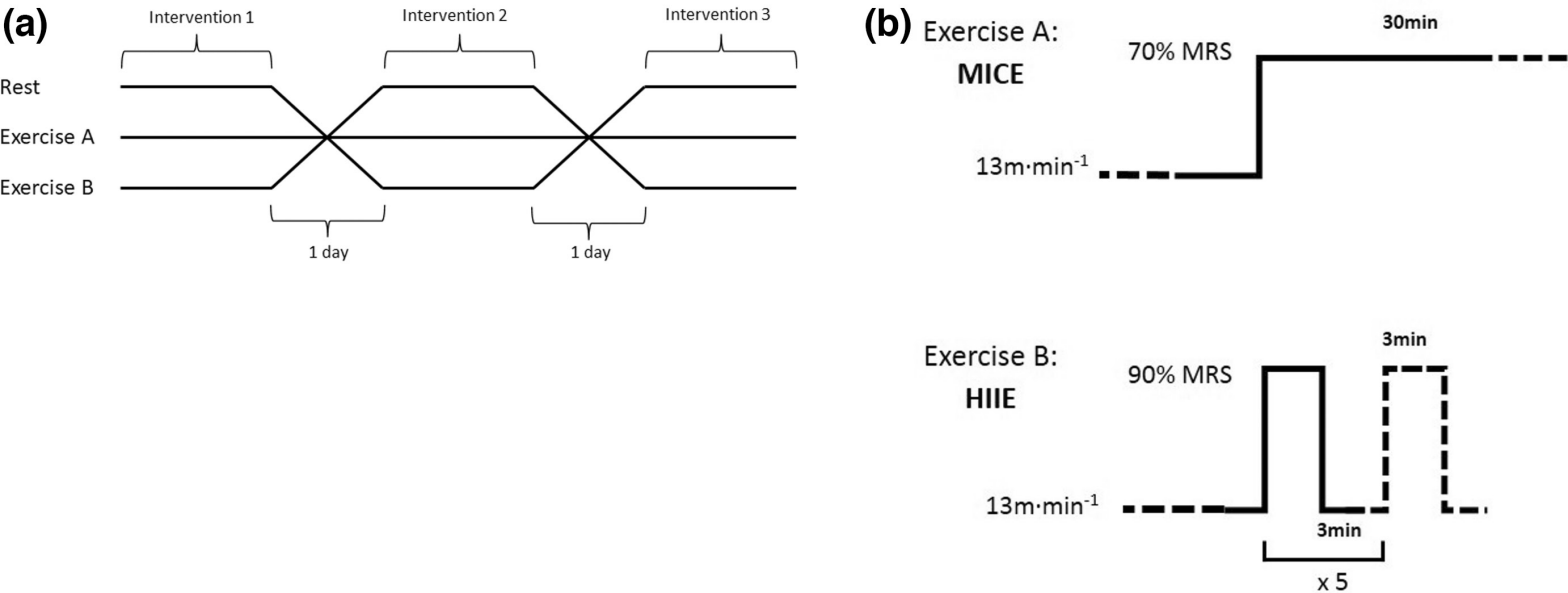
Study design and protocols of exercise. (a) Study design: All animals performed three interventions (rest, exercise A: MICE, exercise B: HIIE) in a randomized order. (b) Schematic representation of the two types of exercise tests.

The animals randomly performed two exercise sessions, as presented in Figure 1, with a 2‐day rest period between each session. The moderate‐intensity continuous exercise (MICE) consisted of running for 30 min at 70% of their PTV, while the high‐intensity intermittent exercise (HIIE) consisted of performing five repetitions of 3 min at 90% of their PTV, interspersed by 3 min of running at 13 m min^−1^. Each session was preceded by a standardized warm‐up of a 10‐min run at 13 m min^−1^. The total duration of each exercise session was similar (i.e., 40 min). Each experimental day was organized as follows. First, animals stayed in a quiet environment, without human presence, for 3 h (from 9 to 12 am). Then, they were handled and placed on the treadmill for a 10‐min environment habituation, immediately followed by the 10‐min warm‐up and the 30‐min exercise session.

#### 2.3.4 Data analysis

**FIGURE 2:**
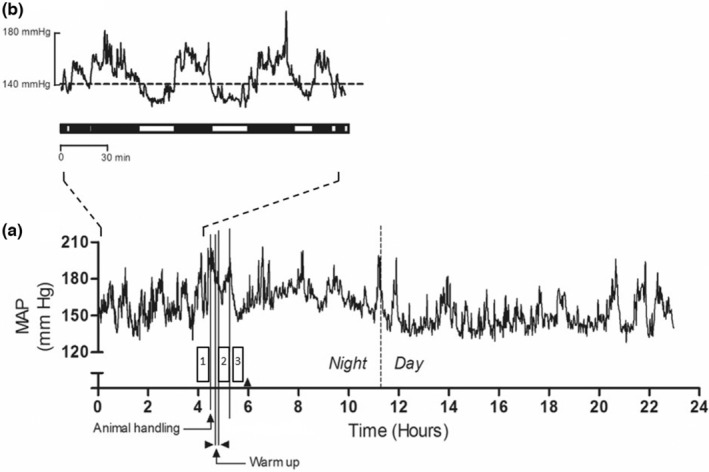
Recording of mean arterial pressure (MAP) during 24 h in a typical SHR. (a): Filled arrowheads indicate a change in acquisition frequency, 1: Pre‐exercise, 2: Exercise, 3: Post‐exercise. The vertical dashed line indicates the end of the night period. (b): MAP 3 h before the exercise period in one SHR. Black horizontal bars below the trace correspond to bursts of MAP increase. N = 13 rats.

For arterial pressure baseline measurement, the baseline value was assessed two ways before exercise (Number 1 in the box, Figure 2.). The baseline 1 (short baseline) value represents the resting value measured 10 min before the experiment (average of three values) when animals are still quiet without human interaction. The baseline 2 (long baseline) value represents the average resting value of the 3 h before the experiment with very little interaction with the animal, which is only possible with a continuous monitoring method like telemetry.

As suggested by Fecchio et al. (2020), post‐exercise hypotension (PEH) was calculated for each rat as follows:

(1) PEHI=Post ExerciseBP−BaselineBP.

(2) PEHII=Post ExerciseBP−Post ControlBP.

(3) $$\begin{array}{c} \mathrm{PEH}\;\mathrm{III}=\left(\text{Post}\;\text{Exercise}\;\mathrm{BP}–\text{Baseline}\;\mathrm{BP}\right)-\\ \;\left(\text{Post}\;\text{Control}\;\mathrm{BP}–\mathrm{Pre}\;\text{Control}\;\mathrm{BP}\right). \end{array}$$

Post‐exercise represents values of arterial pressure obtained 20 min after the exercise session, and post‐ and pre‐control represent values at the same time but on a day without exercise (Table 1). For all equations, we calculated the PEH for each rat considering its exercise and control data (Equations 2 and 3) or its exercise and baseline data (Equation 1). For each condition, the mean represented the average PEH values of the 13 rats.

**TABLE 1:** Methods of measuring the different components of the equations used.

Components of equations	Measurement methods
Baseline	Calculated 10 min before the start of the experiment over 1 min
Post‐exercise	Calculated over 1 min, every 10 min after exercise
Pre‐control	Pre‐exercise values from a non‐experimental day averaged over 1 min at the same time as the Baseline
Post‐control	Post‐exercise values from a non‐experimental day averaged over 1 min at the same time as the Post‐exercise

### 2.4 Statistical analysis

Standard statistical methods were used to calculate means and standard error (SE). The Shapiro–Wilk test verified the normal Gaussian distribution of the data. A one‐way within‐group analysis of variance (ANOVA) was performed to test the null hypothesis that dependent variables were not affected by the exercise protocol.

Multiple comparisons were made using the Newman–Keuls post hoc test. The magnitude of the difference was assessed by Hedge's g for dependent samples. Cohen's scale was used for the interpretation of the effect size. Changes were considered either small (0.20 ≤ |Hedge's g| < 0.50), moderate (0.50 ≤ |Hedge's g| < 0.80), or large (|Hedge's g| ≥ 0.80). The bias and level of agreement between the different methods were assessed using the method described by Bland and Altman (1986). A two‐way repeated measures ANOVA was performed to evaluate the difference between the two types of exercise, looking at the time of onset and the duration of the hypotension. The significance level was set at *p* < 0.05. Statistical tests were conducted using Statistica (Version 10; StatSoft, Tulsa, USA).

## 3 RESULTS

### 3.1 Arterial pressure burst events

**FIGURE 3:**
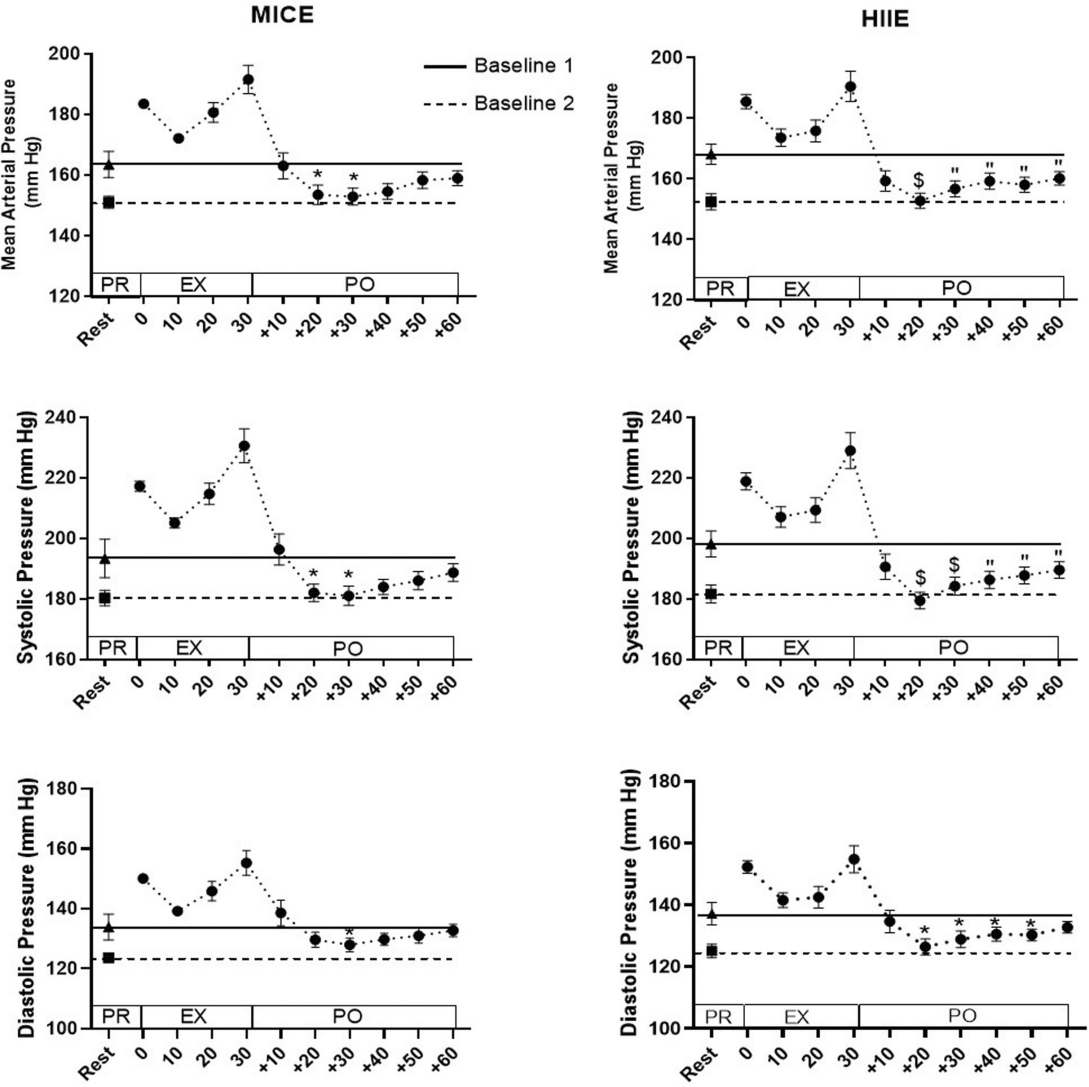
Arterial pressure before, during, and after the two types of exercise. PR: Pre‐exercise, EX: Exercise, PO: Post‐exercise. Baseline 1 (black line) represents the resting value measured 10 min before the experiment, and baseline 2 (dashed line) represents the average resting value 3 h before the experiment. Values are means ± SE for 13 rats. Different from rest value of baseline 1: *p < 0.05, “*p* < 0.01, $*p* < 0.001.

Figure 2a shows an example of the mean arterial pressure (MAP) during 24‐h monitoring, including a 30 min bout of continuous exercise in a typical SHR. MAP was variable, as fluctuations could be observed throughout the observation period. These bursts of MAP variation are shown in the time‐expanded recording in Figure 2b. It should be noted that MAP increased before the start of the exercise due to animal handling and placing on the treadmill. During the 30 min of exercise, we observed an increase in arterial pressure. Once the exercise stopped, arterial pressure decreased. This pattern was detected in all rats, independently of the type of exercise, as presented in Figure 3.

#### 3.1.1 Comparison of the methods used to determine resting BP

**FIGURE 4:**
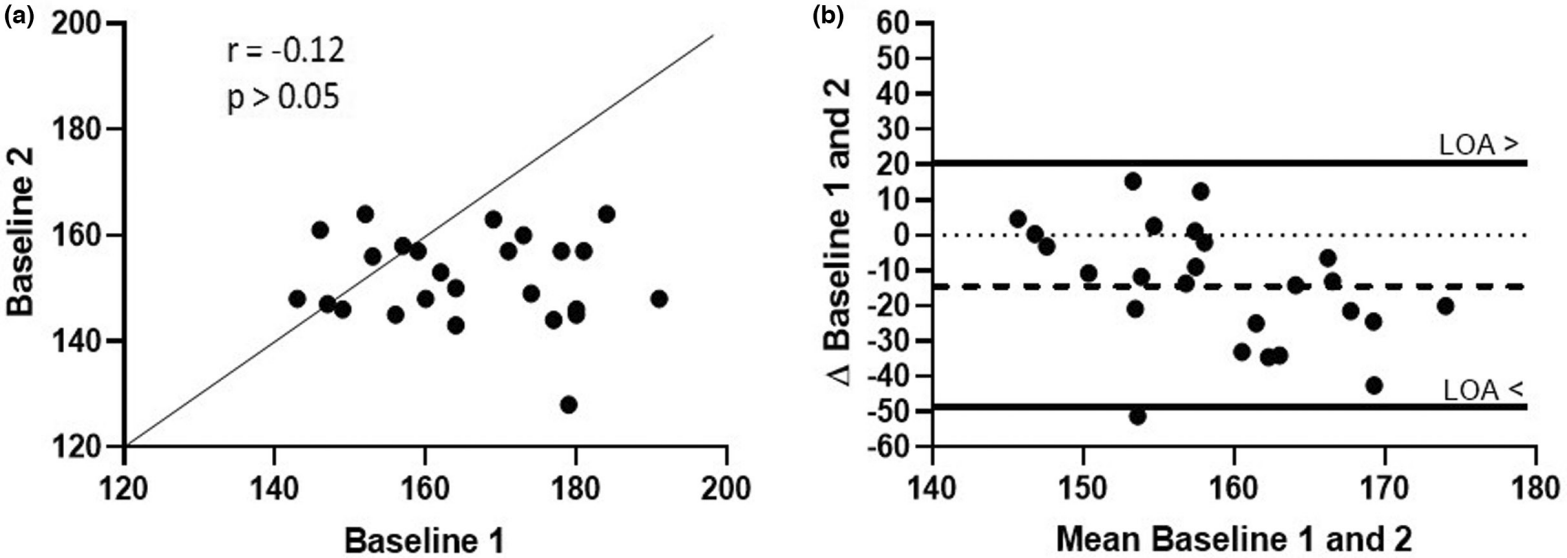
Baseline data analysis. (a) Correlation between baseline 1 and 2, (b) Plot of differences between baseline 1 and 2 versus the mean of the two measurements. The bias of −14 mmHg is represented by the gap between the X axis, corresponding to zero differences, and the parallel dashed line to the *X* axis. N = 13 rats.

The association and the level of agreement between the two methods used to determine the resting BP (i.e., short and long baseline) are shown in Figure 4. Although we found no association between both methods (Figure 4a; *r* = −0.12), an important bias toward lower values was observed when using the long baseline (Figure 4b; −14 ± 17 mmHg, *p* < 0.01, *g* = 1.2) and a 95% limit of agreement of 36 mmHg, which represent 255% of the bias and 23% of the average resting BP.

#### 3.1.2 Comparison of the methods used to determine the magnitude of the PEH

The association and the agreement level between the methods used to determine the PEH magnitude are presented in Table 2. As expected, the magnitude of the PEH was lower when it was calculated using the long baseline (baseline 2) (*p* < 0.05) only with Equations (1) and (3). The association level between these two methods ranged from 0.38 to 0.76, corresponding to a shared variance of 14 to 58%. The systematic error (or bias) ranged from 2 to 9 mmHg, and 95% limits of agreement from 19 to 32 mmHg, which corresponds to 120 to 1298% of the average PEH.

**TABLE 2:** Association and agreement between the different methods of determination of post‐exercise hypotension compiling the two types of exercise

Methods	Mean ± SD^a^	Mean ± SD^b^	*r*	Bias	Hedge's g	95% LOA^c^	
	(mmHg)	(mmHg)		(mmHg)		(mmHg)	%^d^
Computed with baseline 1							
Equation (1) vs. Equation (2)	−11 ± 13	−4 ± 13	0.38	7	0,52	32	475
Equation (2) vs. Equation (3)	−4 ± 13	−13 ± 14	0.51	−9	0,62	31	−340
Equation (1) vs. Equation (3)	−11 ± 13	−13 ± 14	0.71	−2	0,13	24	−1298
Computed with baseline 2							
Equation (1) vs. Equation (2)	3 ± 7*	−3 ± 13	0.59	−7	0,52	23	−120
Equation (2) vs. Equation (3)	−3 ± 13	−2 ± 13*	0.76	2	0,13	19	1045
Equation (1) vs. Equation (3)	3 ± 7	−2 ± 13	0.66	−5	0,43	21	−390

*Note*: *N* = 13 rats.

^a^ Computed from the first equation listed in the Methods column.

^b^ Computed from the second equation listed in the Methods column.

^c^ Limits of agreement (LOA).

^d^ In percentage of the average post‐exercise hypotension.

* Different from mean computed with Baseline 1 with the same equation (*p* < 0.05).

#### 3.1.3 Comparison of MICE and HIIE

**FIGURE 5:**
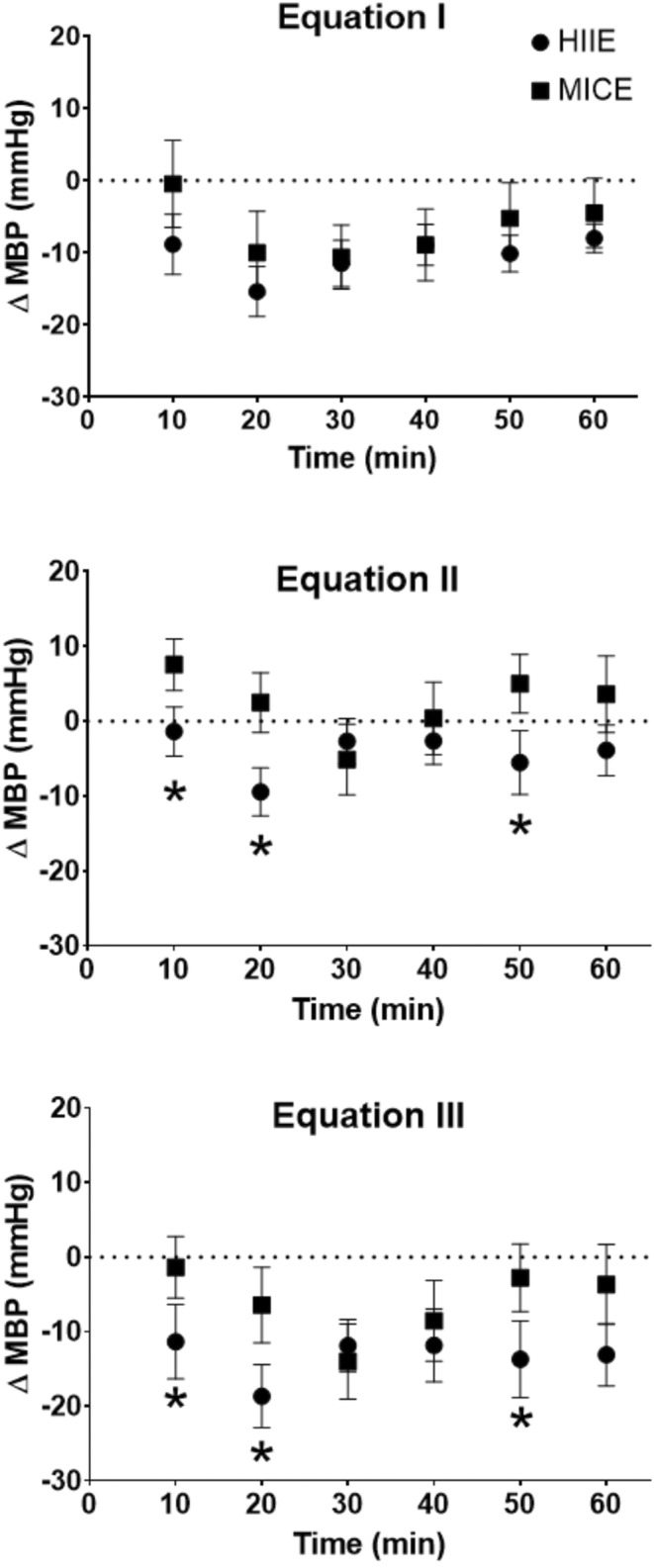
Delta of mean arterial pressure for each exercise according to three methods of measure. The delta was measured with baseline 1. MICE: Moderate‐intensity intermittent exercise, HIIE: High‐intensity intermittent exercise. Equation 1 (PEH I: Up): Post‐exercise ‐ pre‐exercise; equation 2 (PEH II; middle): Post‐exercise ‐ post‐control; equation 3 (PEH III; down): (post‐exercise ‐ pre‐exercise) ‐ (post‐control ‐ pre‐control). Values are means ± SE for 13 rats. Different from MICE value at the same time: *p < 0.05.

BP response to the two types of exercise was quite variable between the 13 animals. Indeed, it can be noted that six rats had hypotension only following HIIE, one animal only following MICE and five rats as a result of both types of exercise. Moreover, we observed in a single rat no PEH with both exercises (Table 3). The PEH computed with each method is presented for each exercise modality in Table 4. We found an important difference in PEH computed with Equation (3) between the two types of exercise, when we look at the MAP (*p* < 0.05), with a larger magnitude of PEH after HIIE. Conversely, no significant differences were observed with the systolic or diastolic pressure. As shown in Figure 5, the kinetics of BP after exercise cessation were different between HIIE and MICE depending of methods used. In fact, we observed some specificities with method II and III, like a PEH that appears sooner (*p* < 0.05) after HIIE (10 min) compared to after MICE (20 and 30 min). Moreover, this was characterized by a longer duration and a larger magnitude (*p* < 0.05). However, no significant differences were observed between the two exercises when values were obtained with method I.

**TABLE 3:** Animals presenting a post‐exercise hypotension

Rats	1	2	3	4	5	6	7	8	9	10	11	12	13
MICE				X	X	X	X	X			X		
HIIE	X	X	X	X	X	X	X	X	X			X	X

*Note*: Animals presenting a post‐exercise hypotension (cross: responder) after two types of exercise, MICE: Moderate intensity continuous exercise and HIIE: High‐intensity intermittent exercise measured with baseline 1.

**TABLE 4:** Post‐exercise hypotension magnitude

		Equation (1)	Equation (2)	Equation (3)
	Mean (mmHg)	−16 ± 5	−7 ± 5	−10 ± 6
MICE	Systolic (mmHg)	−18 ± 6	−6 ± 5	−16 ± 6
	Diastolic (mmHg)	−12 ± 4	−6 ± 5	−8 ± 4
	Mean (mmHg)	−21 ± 3	−12 ± 4	−22 ± 3*
HIIE	Systolic (mmHg)	−23 ± 4	−12 ± 5	−23 ± 5
	Diastolic (mmHg)	−16 ± 3	−11 ± 3	−14 ± 5

*Note*: Equation 1: Post‐exercise ‐ pre‐exercise; equation 2: Post‐exercise ‐ post‐control; equation 3: (post‐exercise ‐ pre‐exercise) ‐ (post‐control ‐ pre‐control); for the two types of exercise (MICE: Moderate intensity intermittent exercise, HIIE: High‐intensity intermittent exercise). Baseline 1 was used in these calculations. Values are means ± SE for 13 rats.

* Different from MICE with the same equation (*p* < 0.05).

## 4 DISCUSSION

This study aimed to compare different methods to assess and apply them to determine the PEH magnitude induced by a HIIE or a MICE. Our main findings were (a) the significant influence of measurement methods on baseline and PEH assessment and (b) the greater magnitude of PEH with baseline 1 after a HIIE in male SHR.

In our study, BP was measured by telemetry, a gold‐standard technique that allows continuous monitoring of freely moving rodents in their home cage. SHR model of hypertension was chosen in this paper since our goal is to explore non‐pharmacological ways (such as physical activity) to reduce blood pressure and it has previously been shown that PEH is more pronounced in hypertensive subjects (Halliwill, 2001). The Baseline values reported in our paper were similar to what was previously published in the literature with male SHR of the same age (Calhoun, Zhu, Chen, & Oparil, 1995; Chandler et al., 1998; da Silva et al., 2008). Nevertheless, our values are lower than those obtained by tail‐cuff (Ibrahim, Berk, & Hughes, 2006; Kubota et al., 2006). Indeed, tail‐cuff can cause stress since it requires animal handling and restraining during measurement, affecting BP's values (Wilde et al., 2017).

When comparing both methods to calculate resting BP values, we found an important difference; baseline 1 provided higher values than baseline 2. This difference mainly originates from the sampling frequency of BP measurements. As shown in Figure 2, BP measured by telemetry revealed an important variability. The same observation has been reported in other telemetry studies (Smith & Gordon, 2005; Williams et al., 2000). This continuous fluctuation in BP is named blood pressure variability (Höcht., 2013; Parati, Vrijens, & Vincze, 2008). It is associated in part with the normal activity of animals (for instance, eating, drinking, and moving) and the autonomic control of BP. Telemetry is the choice method from a physiological point of view since it provides more information than a simple BP time point. Of course, its principal limit is the cost required for telemetry, which may explain the use of less invasive methods such as the tail cuff.

Although it is evident that the determination of baseline BP is an important contributor to the magnitude of the PEH, it is also clear that it is not the only factor. Several calculation methods have been proposed in the literature (Fecchio et al., 2020) and compared in this study, which have advantages and disadvantages. For instance, Equation (1) does not consider time‐related variations (circadian rhythm) as it compares the BP after the exercise bout to the baseline BP measured before the exercise. On the other hand, Equation (2) accounts for the circadian rhythm as it compares values at the same given time on a day without physical exercise. However, the day‐to‐day resting BP variability may influence it. Lastly, Equation (3) controls both confounding factors and would seem to be the best method, but it requires more data for its calculation. When accounting for the systematic error, the 95% limits of agreement and the lack of association between methods, it is clear that they cannot be used interchangeably, even if they use the same baseline (i.e., baseline 1 or baseline 2). This lack of agreement between PEH calculation methods has already been reported in humans after a MICE (Fecchio et al., 2020). However, researchers did not find any differences between pre‐exercise and pre‐control values. In our study, pre‐exercise and pre‐control values were significantly different when the resting value was measured 10 min before the experiment (data not shown), contributing to the poor agreement observed between the PEH calculated from Equations (2) and (3) with baseline 1. Differences appear to be related to sudden changes in BP. This can be explained by the difficulty of controlling all parasite variables, such as noise and animal movements. Indeed, even if the experimenter was not present in the room in both conditions (exercise or rest), external events seem to have disturbed the BP measurements before the exercise. The context is different in humans, where a concordance between Equations (2) and (3) could be observed, notably linked to the fact that the environment is controlled during the measurement and the subject can follow specific guidelines directives during the test.

As previously underscored for baseline determination, the PEH calculation method should be precisely described to allow an appropriate comparison between studies (de Brito et al., 2019). We proposed that the best research methodology to deal with BP variability is to study the BP variability instead of raw values. BP Measurements beat by beat should be made at a time compatible with the storage capacity of the measuring device, for example, 2 h before and 2 h after the “exercise” intervention.

In our study, post‐exercise hypotension was observed only with baseline 1 for both types of exercise modality. This hypotension appears between 20 and 30 min after exercise cessation looking at the systolic and mean arterial BP, depending on exercise modality (i.e., HIIE or MICE). Hypotension after a MICE reported in this paper is in agreement with other articles (Chen, Munch, Quail, & Bonham, 2002; Lee et al., 2009; Minami et al., 2006; Rao, Collins, & DiCarlo, 2002), which highlighted hypotension following a continuous acute exercise, although the time of onset and the delta (25 mmHg vs 16 mmHg in our study, with the same equation) of hypotension are different. These variations can be related to the intensity of continuous exercise. Indeed, the running speed used in the MICE for our study was higher (average of 23 m.min^−1^) than that in other reports. The running speed often used in the literature to measure acute exercise effects is close to our warm‐up speed (Lee et al., 2009; Minami et al., 2006) or lower (Minami et al., 2006; Rao et al., 2002), however, in these studies, the running speed does not seem to have been measured in relative for each animal. To our knowledge, we are the only group that assessed the PEH after a high‐intensity intermittent acute exercise in rodents. However, when analyzing the post‐exercise responses of each rat, we can observe inter‐ and intra‐ individual variabilities. Concerning inter‐individual variability, PEH was not observed in one rat in both types of exercise, but for the intra‐individual variability, some animals present a PEH depending on the type of exercise performed. Of the 12 rats presenting a PEH, five responded to both types of exercise, while six have hypotension only after HIIE. To our knowledge, no publication reported individual results in rats. However, these inter‐individual variations have been observed with continuous moderate and high‐interval training in hypertensive (Lima et al., 2015; Moreira, Cucato, Terra, & Ritti‐Dias, 2016) and non‐hypertensive populations (Bonsu & Terblanche, 2016; Hecksteden, Grutters, & Meyer, 2013). In these studies, subjects are classified as “low” (no PEH) and “high” responders (PEH) following both types of exercise (HIIE and continuous exercise). Moreover, Costa et al. have shown an intra‐individual variation according to exercise type in normotensive patients (Costa et al., 2016).

Finally, if we compare the effects of the two types of exercise within each PEH calculation method, with baseline 1, HIIE seems to cause more marked hypotension than MICE. Indeed, with HIIE, we observed an increase in the delay of the PEH onset (40 min vs. 10 min) in many animals (11 animals vs. 6 with baseline 1) as well as a significantly more marked amplitude of response than for MICE (MAP: −22 ± 3 mmHg vs. −10 ± 6 mmHg with Equation (3), SAP and DAP near significant *p* = 0.07).

To our knowledge, no studies have investigated these effects in hypertensive or non‐hypertensive animals. However, this HIIE hypotensive effect has been reported in hypertensive patients. For example, Ciolac et al, in long‐term‐treated hypertensive patients, showed a similar post‐exercise BP reduction following high‐intensity intermittent and moderate‐intensity continuous acute exercises (Ciolac et al., 2009). In another study, authors demonstrated a greater hypotension magnitude after HIIE compared to MICE on a treadmill in treated hypertensive patients (Pimenta et al., 2019).

In conclusion, the current study demonstrated the importance of the calculation methods in the analysis of the hypotensive effect of a physical exercise to compare the occurrence, magnitude, and duration of PEH between different studies. Moreover, although PEH was observed with both types of exercise, HIIE seems to have a more pronounced hypotensive effect in SHR with a specific calculation approach which is characterized by a longer duration and greater magnitude.

## AUTHORS’ CONTRIBUTIONS

ND, LB, and JL conceived and designed the experiments. AK performed the experiments and analyzed the data. AK, ND, LB, and JL wrote the paper. SB and CC reviewing the first paper. For the revised article, AK, JL, and ND have reviewed the paper and prepared answers for reviewers.

## References

[phy215524-bib-0001] Ambrosetti, M. , Abreu, A. , Corra, U. , Davos, C. H. , Hansen, D. , Frederix, I. , et al. (2020). Secondary prevention through comprehensive cardiovascular rehabilitation: From knowledge to implementation. 2020 update. A position paper from the secondary prevention and rehabilitation section of the European Association of Preventive Cardiology. European Journal of Preventive Cardiology, 2047487320913379. 10.1177/2047487320913379 33611446

[phy215524-bib-0002] Atkinson, G. , Cable, N. T. , & George, K. (2005). The relationship between baseline blood pressure and magnitude of postexercise hypotension. Journal of Hypertension, 23(6), 1271–1272 author reply 1272‐3. 15894905 10.1097/01.hjh.0000170392.92073.33

[phy215524-bib-0003] Bonsu, B. , & Terblanche, E. (2016). The training and detraining effect of high‐ intensity interval training on post‐exercise hypotension in young overweight/obese women. European Journal of Applied Physiology, 116(1), 77–84. 26293124 10.1007/s00421-015-3224-7

[phy215524-bib-0004] Calhoun, D. A. , Zhu, S. T. , Chen, Y. F. , & Oparil, S. (1995). Gender and dietary NaCl in spontaneously hypertensive and Wistar‐Kyoto rats. Hypertension, 26(2), 285–289. 7635536 10.1161/01.hyp.26.2.285

[phy215524-bib-0005] Casonatto, J. , Goessler, K. F. , Cornelissen, V. A. , Cardoso, J. R. , & Polito, M. D. (2016). The blood pressure‐lowering effect of a single bout of resistance exercise: A systematic review and meta‐analysis of randomised controlled trials. European Journal of Preventive Cardiology, 23(16), 1700–1714. 27512052 10.1177/2047487316664147

[phy215524-bib-0006] Chandler, M. P. , Rodenbaugh, D. W. , & DiCarlo, S. E. (1998). Arterial baroreflex resetting mediates postexercise reductions in arterial pressure and heart rate. American Journal of Physiology, 275(5), H1627–H1634. 9815070 10.1152/ajpheart.1998.275.5.H1627

[phy215524-bib-0007] Chen, C. Y. , Munch, P. A. , Quail, A. W. , & Bonham, A. C. (2002). Postexercise hypotension in conscious SHR is attenuated by blockade of substance P receptors in NTS. American Journal of Physiology. Heart and Circulatory Physiology, 283(5), H1856–H1862. 12384463 10.1152/ajpheart.00827.2001

[phy215524-bib-0008] Ciolac, E. G. , Guimaraes, G. V. , VM, D. A. , Bortolotto, L. A. , Doria, E. L. , & Bocchi, E. A. (2009). Acute effects of continuous and interval aerobic exercise on 24‐h ambulatory blood pressure in long‐term treated hypertensive patients. International Journal of Cardiology, 133(3), 381–387. 18501444 10.1016/j.ijcard.2008.02.005

[phy215524-bib-0009] Costa, E. C. , Dantas, T. C. , de Farias Junior, L. F. , Frazao, D. T. , Prestes, J. , Moreira, S. R. , et al. (2016). Inter‐ and intra‐individual analysis of post‐exercise hypotension following a single bout of high‐intensity interval exercise and continuous exercise: A pilot study. International Journal of Sports Medicine, 37(13), 1038–1043. 27676151 10.1055/s-0042-112029

[phy215524-bib-0010] da Silva, A. A. , do Carmo, J. M. , Kanyicska, B. , Dubinion, J. , Brandon, E. , & Hall, J. E. (2008). Endogenous melanocortin system activity contributes to the elevated arterial pressure in spontaneously hypertensive rats. Hypertension, 51(4), 884–890. 18285617 10.1161/HYPERTENSIONAHA.107.100636 PMC2803054

[phy215524-bib-0011] de Brito, L. C. , Fecchio, R. Y. , Pecanha, T. , Lima, A. , Halliwill, J. , & Forjaz, C. L. M. (2019). Recommendations in post‐exercise hypotension: Concerns, best practices and interpretation. International Journal of Sports Medicine, 40(8), 487–497. 31288287 10.1055/a-0938-4415

[phy215524-bib-0012] Dias, D. d. S. , Araujo, A. A. d. , RJC, P. , Bernardes, N. , Sanches, I. C. , & Angelis, K. D. (2017). A dynamic resistance exercise session does not induce post exercise hypotension in female SHR rats. Revista Brasileira de Medicina do Esporte, 23(4), 279–284.

[phy215524-bib-0013] Faria Tde, O. , Targueta, G. P. , Angeli, J. K. , Almeida, E. A. , Stefanon, I. , Vassallo, D. V. , et al. (2010). Acute resistance exercise reduces blood pressure and vascular reactivity, and increases endothelium‐dependent relaxation in spontaneously hypertensive rats. European Journal of Applied Physiology, 110(2), 359–366. 20499250 10.1007/s00421-010-1508-5

[phy215524-bib-0014] Fecchio, R. Y. , Brito, L. C. , Pecanha, T. , & Forjaz, C. L. M. (2020). Post‐exercise hypotension and its hemodynamic determinants depend on the calculation approach. Journal of Human Hypertension, 34(10), 719–726. 31965012 10.1038/s41371-020-0297-5

[phy215524-bib-0015] Gomes Anunciacao, P. , & Doederlein, P. M. (2011). A review on post‐exercise hypotension in hypertensive individuals. Arquivos Brasileiros de Cardiologia, 96(5), e100–e109. 21359479

[phy215524-bib-0016] Halliwill, J. R. (2001). Mechanisms and clinical implications of post‐exercise hypotension in humans. Exercise and Sport Sciences Reviews, 29(2), 65–70. 11337825 10.1097/00003677-200104000-00005

[phy215524-bib-0017] Hecksteden, A. , Grutters, T. , & Meyer, T. (2013). Association between postexercise hypotension and long‐term training‐induced blood pressure reduction: A pilot study. Clinical Journal of Sport Medicine, 23(1), 58–63. 22673537 10.1097/JSM.0b013e31825b6974

[phy215524-bib-0018] Höcht, C. (2013). Blood pressure variability: Prognostic value and therapeutic implications. ISRN Hypertension, 2013, 1–16.

[phy215524-bib-0019] Ibrahim, J. , Berk, B. C. , & Hughes, A. D. (2006). Comparison of simultaneous measurements of blood pressure by tail‐cuff and carotid arterial methods in conscious spontaneously hypertensive and Wistar‐Kyoto rats. Clinical and Experimental Hypertension, 28(1), 57–72. 16443565 10.1080/10641960500386817

[phy215524-bib-0020] Jones, H. , George, K. , Edwards, B. , & Atkinson, G. (2007). Is the magnitude of acute post‐exercise hypotension mediated by exercise intensity or total work done? European Journal of Applied Physiology, 102(1), 33–40. 17879098 10.1007/s00421-007-0562-0

[phy215524-bib-0021] Kubota, Y. , Umegaki, K. , Kagota, S. , Tanaka, N. , Nakamura, K. , Kunitomo, M. , & Shinozuka, K. (2006). Evaluation of blood pressure measured by tail‐cuff methods (without heating) in spontaneously hypertensive rats. Biological and Pharmaceutical Bulletin, 29(8), 1756–1758. 16880638 10.1248/bpb.29.1756

[phy215524-bib-0022] Lee, S. K. , Kim, C. S. , Kim, H. S. , Cho, E. J. , Joo, H. K. , Lee, J. Y. , Lee, E. J. , Park, J. B. , & Jeon, B. H. (2009). Endothelial nitric oxide synthase activation contributes to post‐exercise hypotension in spontaneously hypertensive rats. Biochemical and Biophysical Research Communications, 382(4), 711–714. 19306842 10.1016/j.bbrc.2009.03.090

[phy215524-bib-0023] Lima, A. H. , Miranda, A. S. , Correia, M. A. , Soares, A. H. , Cucato, G. G. , Sobral Filho, D. C. , et al. (2015). Individual blood pressure responses to walking and resistance exercise in peripheral artery disease patients: Are the mean values describing what is happening? Journal of Vascular Nursing, 33(4), 150–156. 26567054 10.1016/j.jvn.2015.09.001

[phy215524-bib-0024] Lizardo, J. H. , Silveira, E. A. , Vassallo, D. V. , & Oliveira, E. M. (2008). Post‐resistance exercise hypotension in spontaneously hypertensive rats is mediated by nitric oxide. Clinical and Experimental Pharmacology and Physiology, 35(7), 782–787. 18430048 10.1111/j.1440-1681.2008.04950.x

[phy215524-bib-0025] MacDonald, J. R. (2002). Potential causes, mechanisms, and implications of post exercise hypotension. Journal of Human Hypertension, 16(4), 225–236. 11967715 10.1038/sj.jhh.1001377

[phy215524-bib-0026] Marik, P. E. , & Rivera, R. (2011). Hypertensive emergencies: an update. Current Opinion in Critical Care, 17(6), 569–580. 21986463 10.1097/MCC.0b013e32834cd31d

[phy215524-bib-0027] Meissner, A. (2016). Hypertension and the Brain: A risk factor for more than heart disease. Cerebrovascular Diseases, 42(3–4), 255–262. 27173592 10.1159/000446082

[phy215524-bib-0028] Melo, C. M. , Alencar Filho, A. C. , Tinucci, T. , Mion, D., Jr. , & Forjaz, C. L. (2006). Postexercise hypotension induced by low‐intensity resistance exercise in hypertensive women receiving captopril. Blood Pressure Monitor, 11(4), 183–189. 10.1097/01.mbp.0000218000.42710.91 16810028

[phy215524-bib-0029] Minami, N. , Mori, N. , Nagasaka, M. , Ito, O. , Kurosawa, H. , Kanazawa, M. , et al. (2006). Mechanism behind augmentation in baroreflex sensitivity after acute exercise in spontaneously hypertensive rats. Hypertension Research, 29(2), 117–122. 16755145 10.1291/hypres.29.117

[phy215524-bib-0030] Moreira, S. R. , Cucato, G. G. , Terra, D. F. , & Ritti‐Dias, R. M. (2016). Acute blood pressure changes are related to chronic effects of resistance exercise in medicated hypertensives elderly women. Clinical Physiology and Functional Imaging, 36(3), 242–248. 25524237 10.1111/cpf.12221

[phy215524-bib-0031] Parati, G. , Vrijens, B. , & Vincze, G. (2008). Analysis and interpretation of 24‐h blood pressure profiles: Appropriate mathematical models may yield deeper understanding. American Journal of Hypertension, 21(2), 123–125 discussion 127‐9. 18268479 10.1038/ajh.2007.27

[phy215524-bib-0032] Pimenta, F. C. , Montrezol, F. T. , Dourado, V. Z. , da Silva, L. F. M. , Borba, G. A. , de Oliveira, V. W. , et al. (2019). High‐intensity interval exercise promotes post‐ exercise hypotension of greater magnitude compared to moderate‐intensity continuous exercise. European Journal of Applied Physiology, 119(5), 1235–1243. 30848358 10.1007/s00421-019-04114-9

[phy215524-bib-0033] Rao, S. P. , Collins, H. L. , & DiCarlo, S. E. (2002). Postexercise alpha‐adrenergic receptor hyporesponsiveness in hypertensive rats is due to nitric oxide. American Journal of Physiology. Regulatory, Integrative and Comparative Physiology, 282(4), R960–R968. 11893598 10.1152/ajpregu.00490.2001

[phy215524-bib-0034] Rapsomaniki, E. , Timmis, A. , George, J. , Pujades‐Rodriguez, M. , Shah, A. D. , Denaxas, S. , White, I. R. , Caulfield, M. J. , Deanfield, J. E. , Smeeth, L. , Williams, B. , Hingorani, A. , & Hemingway, H. (2014). Blood pressure and incidence of twelve cardiovascular diseases: Lifetime risks, healthy life‐years lost, and age‐specific associations in 1.25 million people. Lancet, 383(9932), 1899–1911. 24881994 10.1016/S0140-6736(14)60685-1 PMC4042017

[phy215524-bib-0035] Reger, P. O. , Kolwicz, S. C. , & Libonati, J. R. (2012). Acute exercise exacerbates ischemia‐induced diastolic rigor in hypertensive myocardium. Springerplus, 1, 46. 23961371 10.1186/2193-1801-1-46 PMC3725917

[phy215524-bib-0036] Smith, E. G. , & Gordon, C. J. (2005). The effects of Chlorpyrifos on blood pressure and temperature regulation in spontaneously hypertensive rats. Basic Clinical Pharmacology Toxicology, 96(6), 503–511. 15910416 10.1111/j.1742-7843.2005.pto_15.x

[phy215524-bib-0037] Sosner, P. , Gayda, M. , Dupuy, O. , Garzon, M. , Lemasson, C. , Gremeaux, V. , Lalongé, J. , Gonzales, M. , Hayami, D. , Juneau, M. , Nigam, A. , & Bosquet, L. (2016). Ambulatory blood pressure reduction following high‐intensity interval exercise performed in water or dryland condition. Journal of the American Society of Hypertension, 10(5), 420–428. 27026570 10.1016/j.jash.2016.02.011

[phy215524-bib-0038] Taylor, C. E. , Jones, H. , Zaregarizi, M. , Cable, N. T. , George, K. P. , & Atkinson, G. (2010). Blood pressure status and post‐exercise hypotension: An example of a spurious correlation in hypertension research? Journal of Human Hypertension, 24(9), 585–592. 20054347 10.1038/jhh.2009.112

[phy215524-bib-0039] Wilde, E. , Aubdool, A. , Thakore, P. , Baldissera, L. , Alawi, K. , Keeble, J. , Nandi, M. , & Brain, S. (2017). Tail‐cuff technique and its influence on central blood pressure in the mouse. Journal of American Heart Association., 6, e005204. 10.1161/JAHA.116.005204 PMC5669161 28655735

[phy215524-bib-0040] Williams, T. D. , Chambers, J. B. , May, O. L. , Henderson, R. P. , Rashotte, M. E. , & Overton, J. M. (2000). Concurrent reductions in blood pressure and metabolic rate during fasting in the unrestrained SHR. The American Journal of Physiology‐Regulatory, Integrative and Comparative Physiology, 278(1), R255–R262. 10644647 10.1152/ajpregu.2000.278.1.R255

